# Supplementary material for: Feasibility of using chest computed tomography (CT) imaging at the first lumbar vertebra (L1) level to assess skeletal muscle mass: a retrospective study
Source: PeerJ. 2023 Dec 11;11:e16652. doi: 10.7717/peerj.16652 (PMC10720423; doi:10.7717/peerj.16652)
Supplement: Supplemental Information 3 — A: The association between L1 SMI and L3 SMI. B: The association in men and women, respectively. C: The association between L1 SMA and L3 SMA.D: The association in men and women, respectively. Abbreviations: L1, the first lumbar vertebra; L3, the third lumbar vertebra; SMI, skeletal muscle index. [file peerj-11-16652-s003.docx]

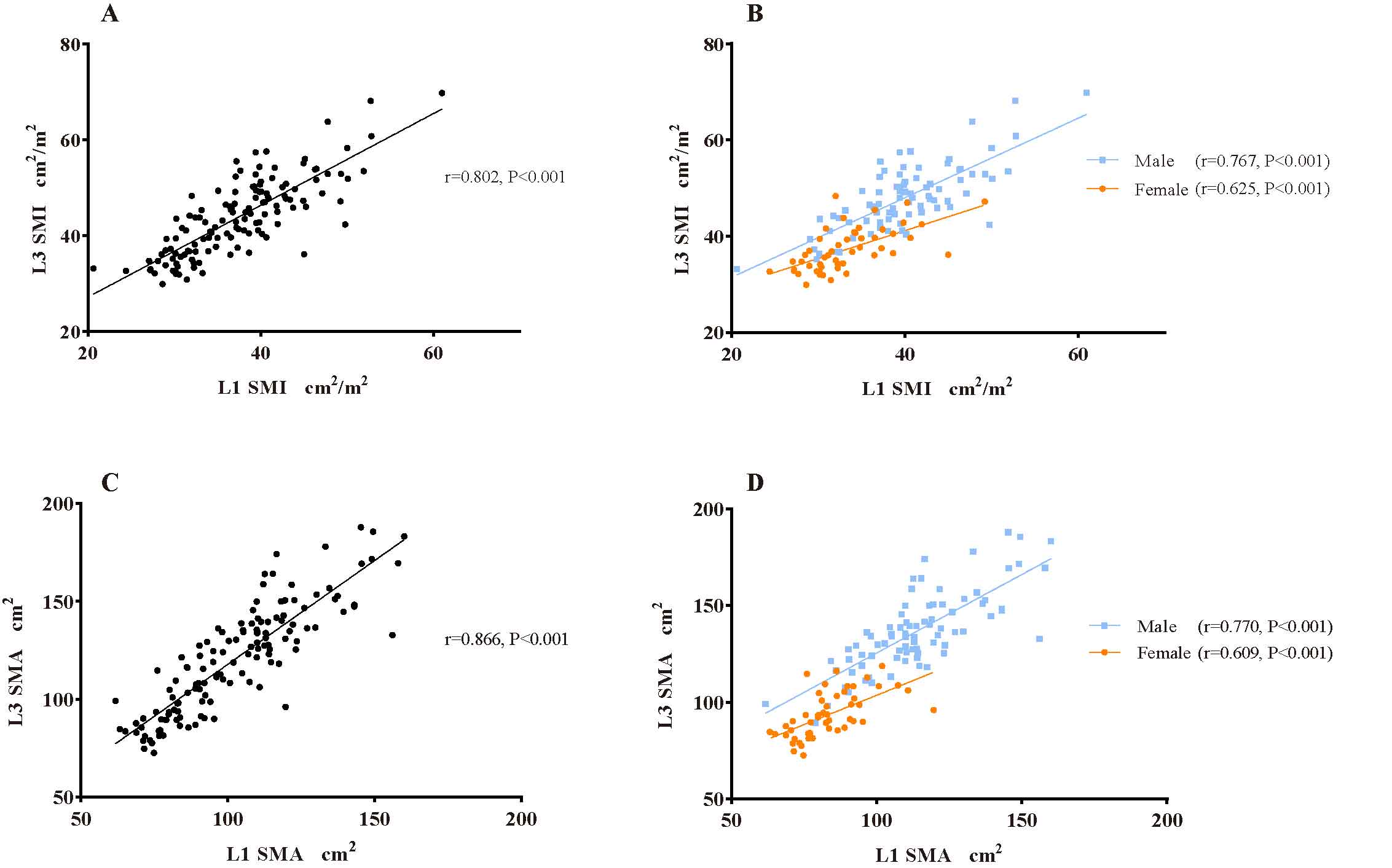


Title: The association between L1 SMI/SMA and L3 SMI/SMA.

Legend:

**A**: The association between L1 SMI and L3 SMI. **B**: The association in men and women, respectively. **C**: The association between L1 SMA and L3 SMA. **D**: The association in men and women, respectively.

Abbreviations: L1, the first lumbar vertebra; L3, the third lumbar vertebra; SMI, skeletal muscle index.
